# Influence of Zr Addition on the Microstructure and Hydrogenation Kinetics of Ti_50−x_V_25_Cr_25_Zr_x_ (x = 0, 5, 7, and 9) Alloys

**DOI:** 10.3390/ma17061366

**Published:** 2024-03-16

**Authors:** Qianying Zeng, Feng Wang, Zhengxi Li, Maohua Rong, Jiang Wang, Zhongmin Wang

**Affiliations:** 1 School of Materials Science and Engineering, Guilin University of Electronic Technology, Guilin 541000, China; 2 Guangxi Academy of Sciences, Nanning 530007, China

**Keywords:** Ti_50_V_25_Cr_25_ hydrogen storage alloys, Zr element substitution, microstructure, hydrogen absorption/desorption properties

## Abstract

Due to the poor activation performance and kinetics of Ti_50_V_25_Cr_25_ alloys, the element Zr was added to improve the phase structure of the alloy and achieve a high-performance hydrogen storage alloy. The Ti_50−x_V_25_Cr_25_Zr_x_ (x = 0, 5, 7, and 9) system alloys were prepared by arc melting. The alloys were analyzed using an X-ray diffractometer (XRD), scanning electron microscope (SEM), and differential scanning calorimeter (DSC). The hydrogen storage capabilities of the alloys were also obtained by the Sievert volumetric method. The results indicated that the alloy with Zr added had a combination of the C15 Laves phase and the BCC phase, whereas the Zr-free alloy had a BCC single phase. The partial replacement of Zr with Ti resulted in an increase in the lattice parameters of the main phase. The hydrogen storage kinetic performance and activation of the alloys both significantly improved with an increasing Zr concentration. The time to reach 90% of the maximum hydrogen storage capacity decreased to 2946 s, 230 s, and 120 s, respectively, with the increases in Zr concentration. The initial hydrogen absorption content of the alloys increased and then decreased after the addition of the element Zr. The second phase expanded with an increasing Zr concentration, which in turn decreased the abundance of the BCC main phase. The Ti_43_V_25_Cr_25_Zr_7_ alloy showed good cycle stability and hydrogen-desorption performance, and it could absorb 90% of the maximum hydrogen storage capacity in around 230 s. The maximum hydrogen-absorption capacity of the alloy was 2.7 wt%. The diffusion activation energy of hydrogen desorption dropped from 102.67 kJ/mol to 92.62 kJ/mol.

## 1. Introduction

With depleting fossil energy reserves, renewable energy sources have become critical. Hydrogen energy has received extensive attention as a sustainable energy source due to its great efficiency and environmental friendliness [1]. The utilization of hydrogen energy involves its preparation, storage, and transportation. The storage and distribution of hydrogen is a critical aspect of the industrialization of hydrogen energy [2,3,4,5]. Owing to their ability to store hydrogen safely, their comparatively large hydrogen storage capacity, and their relatively low hydrogen pressure, hydrogen storage alloys have the potential to displace existing hydrogen storage systems such as high-pressure gas storage and cryogenic liquid storage [2,6,7,8,9]. The Ti-V solid solution has a potential hydrogen storage content of 3.7 wt%, which is higher than that of AB_5_ and AB_2_ alloys, and it has milder hydrogen-absorption conditions than Mg-based alloys [10,11,12]. Therefore, Ti-V-Cr system alloys have been widely studied as third-generation hydrogen storage alloys [13,14,15,16]. The Ti-V-Cr alloys in the Ti-V system have a lower hysteresis on the pressure composition isotherm (PCT) plateau than Ti-V-Mn alloys, which are promising candidates [17]. However, the use of Ti-V-Cr alloys is limited due to their poor activation, low PCT plateau, and low dehydrogenation pressure plateau for monohydride [18,19,20]. Studies have shown that alloying is one of the most effective methodologies to improve the properties of Ti-V-Cr alloys [21,22].

In recent years, research on the poor activation of pure body-center cubic (BCC) phase alloys has increasingly focused on the introduction of transition metals to produce Laves phases to improve their properties. Kamble et al. [23,24] investigated the effect of 4 wt% Zr on 52Ti-12V-36Cr alloys. They reported that the incubation time depended on the average particle size rather than the particle size distribution. When the average particle size was less than 0.5 mm, it took only 1 min to reach 95% of the maximum hydrogen-absorption capacity, which greatly reduced the activation time of the alloy. In contrast, the first hydrogen absorption of the alloy without Zr takes about 5 min. Feng Zhenyu et al. [25] found that the addition of Zr improved the hydrogen-absorption capacities and activation properties of the alloy to about 1.8 wt% at room temperature, but decreased the hydrogen-desorption properties. Studies by Salma Sleiman et al. [7] involved the addition of different proportions of Zr to the Ti_1_V_0.9_Cr_1.1_ alloy, and the results showed that the alloy had a rapid kinetic performance and high hydrogen storage capacity when Zr = 4 wt%. Hangzhou Ming et al. [26] published a paper in which they studied the effect of Zr substitution on the Ti_20−x_Zr_x_Cr_24_Mn_8_V_40_Fe_8_ (x = 0, 1, 2, 3, and 4) alloys and found that the addition of Zr resulted in alloys composed of the BCC and C14 Laves phases. After adding 4 wt% of Zr, the hydrogen absorption reached a maximum content of 2.38 wt%, with excellent kinetic performance. In another experiment [27], they studied the substitution of Zr for V in Ti_10_V_84_Fe_6_ and found that the second phase abundance increased with an increasing Zr content. The activation performance improved, but the hydrogen absorption and desorption amount decreased by adding only 1 at% of Zr. K. Shashikala et al. [28] found that, by substituting Zr for Ti in TiVCr alloys, the alloy with 5 at% of Zr had the highest hydrogen storage of 3.53 wt%, with a small plateau hysteresis and improved cycling performance. According to Daniela Bellon Monsalve et al. [29], the alloy had a maximum hydrogen uptake of 4.2 wt% and was able to absorb it completely within 10 min at x = 6 wt%. However, increasing the amount of x resulted in a decrease in the hydrogen absorption of the alloy, which was related to the amount of Zr_3_Fe added. At x = 8 wt%, the crystallite size decreased. Asheesh Kumar et al. [30] investigated the effect of Al on Ti_50_Cr_25_V_25_ by synthesizing Ti_2−x_ CrVAl_x_ (x = 0.05, 0.1) alloys, and found that the PCT plateau pressure increased gradually with increasing Al content, and the alloys had a maximum hydrogen uptake of 3.9 wt% for x = 0.05.

This work focuses on the Ti_50_V_25_Cr_25_ alloy in the Ti-V-Cr alloy series, which shows higher hydrogen storage capacities, hydrogen absorption and desorption under moderate conditions, and low plateau hysteresis. However, the low plateau pressure, high hydrogen release temperature, and poor activation behavior of the alloy hinder its applications [31,32,33,34]. Based on the Ti_50_V_25_Cr_25_ alloy formulation, the goals of the present work are to improve its activation performance and reduce the hydrogen release temperature by adding varied Zr contents, and to find the optimal amount to add.

## 2. Materials and Methods

The experimental materials included Ti, V, Cr, and Zr (except for Zr, which has a purity of 99.95%, the others have purities of 99.99%), and these materials were all supplied by ZhongNuo Advanced Material (Beijing) Technology Co., Ltd. (Beijing, China). The raw materials were weighed according to the stoichiometric ratio by a non-consumable vacuum arc melting furnace with water cooling under the protection of argon gas. In order to ensure the homogeneity of the ingots, the ingots were turned over and remelted four times after water cooling, and then the ingots were taken out after the ingots cooled. The experimental ingots were prepared in the air by mechanical crushing. It is worth noting that the ingots for XRD testing needed to be sieved through a 400-mesh sieve, while the remaining ingots were milled to approximately a 200-mesh size.

The crystal structure was determined by an X-ray diffractometer X’Pert-PROX (Bruker, Karlsruhe, Germany, Cu Kα source, step size 0.02°, scanning range 20°~90°), supported by JADE 9 for analysis, and refined by GSASII. The microstructures and morphologies of the specimens were analyzed by scanning electron microscopy (SEM, Quanta 450 FEG, FEI, Hillsboro, OR, USA). The dehydrogenation temperature of the alloy was observed using a TASDT-Q600 synchronous DSC-TGA analyzer (Netzsch, Selb, Germany) under argon flow. The experiment was heated from room temperature to 823 K with ramp rates of 5, 10, 15, and 20 K/min. The hydrogen storage performance was evaluated by a homemade Sievert-type hydrogen storage device (model FINESORB-3110, Zhejiang Finetec Instruments Co., Ltd., Hangzhou, China). Approximately 0.3 g of sample was wrapped into a stainless-steel reactor cell, and then argon was purged through the device for leak detection and gas calibration. Hydrogen absorption tests were conducted at 3 MPa of high-purity H_2_ following dynamic vacuum processing for 2 h at 673 K and subsequent cooling to room temperature. The alloy sample was saturated with hydrogen, after which it was desorbed from the sample at 673 K for 2 h. PCT tests were performed after activation at 303 K, 333 K, and 363 K.

## 3. Results and Discussion

### 3.1. Structural Characterizations

**Figure 1:**
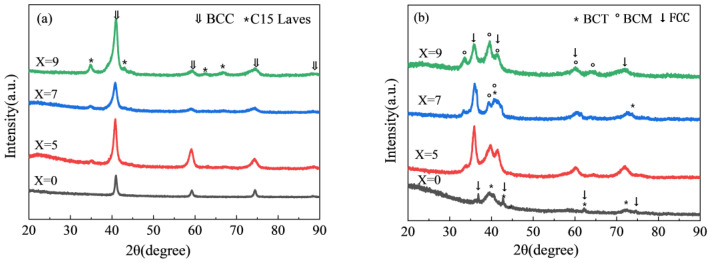
XRD patterns of Ti_50−x_V_25_Cr_25_Zr_x_ (x = 0, 5, 7, and 9) alloys before and after hydrogen absorption: (**a**) as-cast alloys; (**b**) after hydrogen absorption.

The alloy was removed by arc melting, then milled in an agate mortar, and finally filtered through a 400-mesh sieve, and the alloy powder that met this requirement was used for XRD investigation. Figure 1a shows the XRD pattern of the Ti_50−x_V_25_Cr_25_Zr_x_ (x = 0, 5, 7, and 9) alloy system, where it is observed that only the BCC single phase exists when x = 0. After adding the Zr element, the alloy is composed of the BCC primary phase and the C15 Laves phase. The proportion of the secondary phase increases with Zr addition, which is consistent with slight left shifts, and the lattice parameter of the BCC phase main peak increases, due to the atomic radius of Zr (160 pm) which is larger than that of Ti (147 pm). The XRD pattern after hydrogen absorption is shown in Figure 1b. At 303 K, the Ti_50_V_25_Cr_25_ alloy, after saturation hydrogen absorption, shows monohydride with a body-centered tetragonal (BCT) structure (V_2_H, JCPDS#97-065-3722) and dihydride with a face-centered cubic (FCC) structure (VH_2_, JCPDS#97-016-4603). The addition of the Zr element results in the alloy being composed of a base-centered monoclinic (BCM) phase (ZrVH_1.18_, JCPDS#97-008-8252) and an FCC phase (Ti_0.35_V_0.65_H_1.95_, JCPDS#04-007-8401) after saturation hydrogen absorption. The Ti_43_V_25_Cr_25_Zr_7_ alloy is composed of three phases. The substitution of Zr for Ti creates different types of interstitial space, resulting in distinct hydride phases due to the different surrounding environments [35].

**Figure 2:**
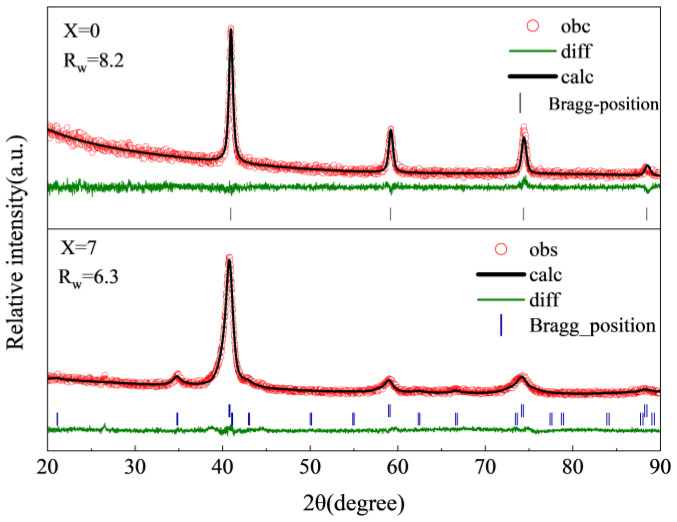
Rietveld refinement mapping of XRD of as-cast Ti_50−x_V_25_Cr_25_Zr_x_ (x = 0 and 7) alloys.

Taking the Ti_50−x_V_25_Cr_25_Zr_x_ (x = 0 and 7) alloys as an example, they were refined using GSASII [36]. The refined patterns and results of the Ti_50−x_V_25_Cr_25_Zr_x_ (x = 0 and 7) alloys are shown in Figure 2 and Table 1, respectively. It is observed that the lattice constant of the BCC phase increases from the initial 3.128 Å to 3.133 Å due to the larger metallic radius of Zr than that of Ti. In addition, the phase abundance of the BCC phase was decreased from 100% to 85.9%, with the Laves phase accounting for 14.1% at this point.

**Figure 3:**
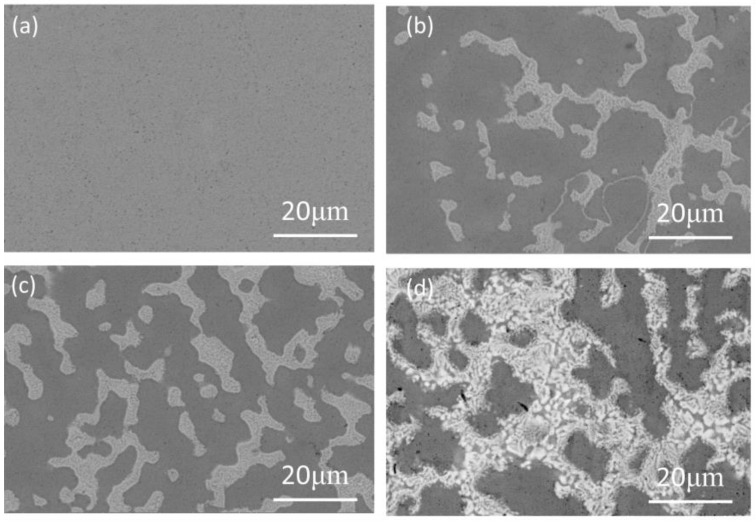
SEM images of as-cast Ti_50−x_V_25_Cr_25_Zr_x_ alloys: (**a**) x = 0; (**b**) x = 5; (**c**) x = 7; (**d**) x = 9.

The microstructures of the Ti_50−x_V_25_Cr_25_Zr_x_ (x = 0, 5, 7, and 9) alloys are shown in Figure 3. The Ti_50_V_25_Cr_25_ hydrogen storage alloy displays a single-phase BCC structure, while the Zr-containing samples contain two phases, consistent with the XRD analysis results. In general, elements with relatively large atomic numbers appear lighter in SEM images [6]. After Zr addition, the Ti_50−x_V_25_Cr_25_Zr_x_ (x = 5, 7, and 9) alloys exhibit a striking, gray second phase. According to Table 2, an increasing trend in the presence of the elements V and Zr can be observed in the BCC phase. The V and Zr content reach a maximum of 30.14 at% and 3.3 at% for x = 9 at%, respectively. Furthermore, the content of Zr gradually increases in the BCC phase and C15 Laves phase, with a peak of 20.61 at% in the secondary phase. The substitution of Zr for Ti partially has the potential to amplify the cell volume and lattice parameter of the BCC phase. The BCC phase of the alloy changes from dendritic to columnar and gradually refines, which is attributed to the increase in Zr, as evident from Figure 3c,d. In addition, the light gray phase increases as we go from Zr to Ti substitution, which indicates that the Zr element is a promoter of secondary phase formation. The secondary phase, which allows hydrogen atoms to penetrate the matrix, tends to generate new cracks at the boundaries of the BCC phase in the early stages, increasing the hydrogen-absorbing surface area and accelerating the hydrogen-absorption rate [27,37].

**Figure 4:**
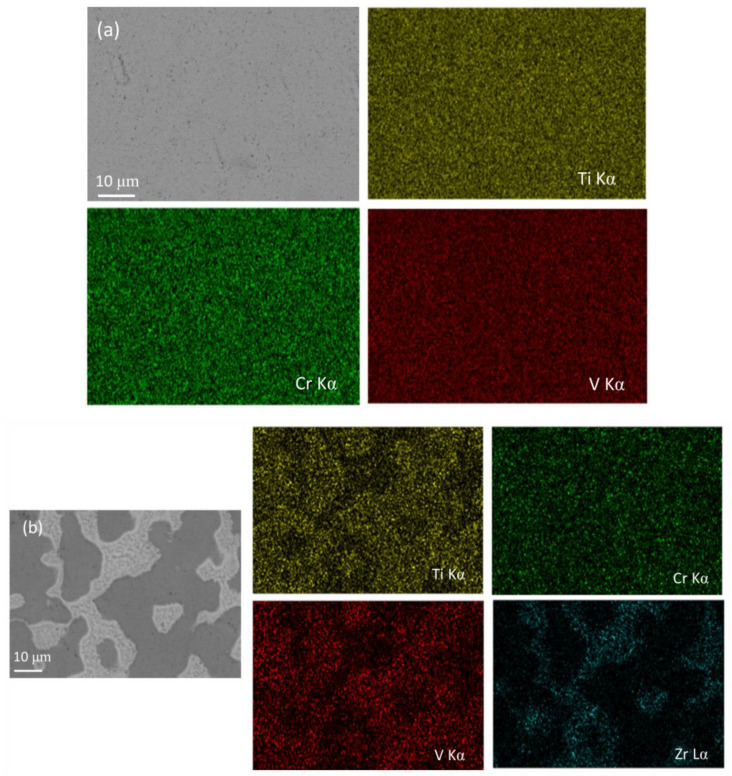
Elements mapping of as-cast Ti_50−x_V_25_Cr_25_Zr_x_ alloys: (**a**) x = 0; (**b**) x = 7.

Two samples of the Ti_50−x_V_25_Cr_25_Zr_x_ (x = 0 and 7) alloys were subjected to surface scans, and the results are shown in Figure 4. The Ti_50_V_25_Cr_25_ alloy demonstrates a BCC single phase with a uniform distribution of Ti, V, and Cr elements. A second phase occurs in the Ti_43_V_25_Cr_25_Zr_7_ alloy in which the element Cr is uniformly distributed and the Ti and V contents of the main phase are higher than those of the second phase. Almost all the Zr is concentrated in the C15 Laves phase, which is consistent with the EDS results (EDS diagrams for Zr = 5 are in the Supplementary Materials).

### 3.2. Absorption and Desorption Properties

**Figure 5:**
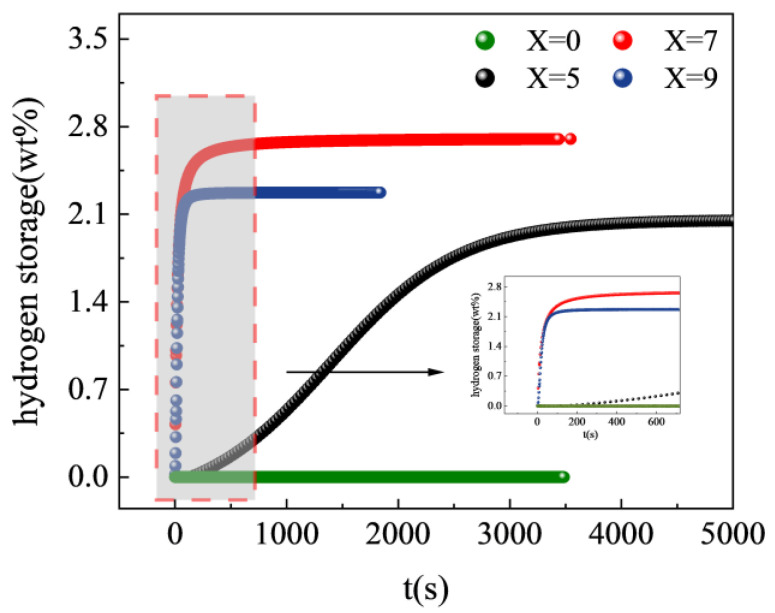
First hydrogen absorption curves of Ti_50−x_V_25_Cr_25_Zr_x_ (x = 0, 5, 7, and 9) alloys.

The kinetic properties of hydrogen storage alloys are a crucial criterion for their practical application. When an alloy is milled, it is exposed to air, and an oxide film forms on its surface, which affects its activation performance. Approximately 0.5 g of the alloy sample was milled to a 200-mesh size. Then, 0.3 g of the alloy powder was weighed and wrapped with a nickel mesh before being placed in a stainless-steel reactor for testing. The alloy was pretreated and tested for hydrogen absorption at 3 MPa H_2_ and 303 K. The image displaying the results is shown in Figure 5. The figure shows that the Ti_50_V_25_Cr_25_ alloy cannot absorb hydrogen after pretreatment and the substitution of Zr elements decreases the incubation time of the alloy. In the first hydrogen absorption tests, the maximum hydrogen absorptions of the Ti_50−x_V_25_Cr_25_Zr_x_ (x = 0, 5, 7, and 9) alloys were 0 wt%, 2.04 wt%, 2.7 wt%, and 2.27 wt%, respectively. It is evident that the inclusion of Zr effectively improves the hydrogen-absorption kinetics of the alloy, while the maximum hydrogen absorption capacity shows an increasing and then decreasing trend. The analysis aims to identify an increase in the abundance of the C15 Laves phase and a decrease in the abundance of the primary hydrogen-absorbing phase. Additionally, the decrease in hydrogen storage capacity is attributed to the partial replacement of Ti by Zr in the BCC phase, which reduces the number of Ti-rich tetrahedral interstitials. After 3000 s, the Ti_45_V_25_Cr_25_Zr_5_ alloy has absorbed 2.04 wt% of hydrogen, which indicates an improved kinetic performance compared to the ternary alloy. However, the hydrogen-absorption capacity in the first test did not reach full activation, as judged by the trend in hydrogen absorption. As the Zr content increases, the time taken for the alloy to reach 90% of the total hydrogen absorption decreases to 2946 s, 230 s, and 120 s, respectively. This phenomenon can be attributed to changes in the composition or thickness of the alloy oxide film after the addition of Zr [26]. Zirconium (Zr) has a higher affinity for oxygen than other elements in the alloy. Therefore, the addition of Zr changes the composition of the oxide layer on the surface of the alloy, reducing the oxygen concentration and the thickness of the oxide layer. This change facilitates the entry of hydrogen atoms into the interior of the alloy. As a result, hydrogen atoms can pass more easily through the oxide film on the metal surface and into the matrix [38]. Furthermore, a greater abundance of the second phase and an increase in channels for hydrogen atoms to penetrate the alloy interior contributed to an increase in the Zr content. The higher the Zr content, the faster the rate of hydrogen diffusion into the alloy. Based on the amount and rate of hydrogen absorption, the Ti_43_V_25_Cr_25_Zr_7_ alloy demonstrates the best hydrogen storage properties.

**Figure 6:**
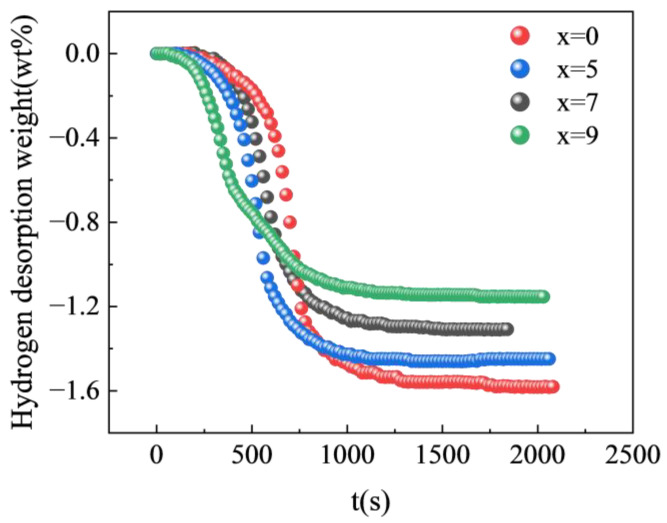
Kinetics curves of hydrogen desorption of Ti_50−x_V_25_Cr_25_Zr_x_ (x = 0, 5, 7, and 9) alloys at 673 K.

The effect of the Zr element on the hydrogen storage performance of alloys is investigated in this study, and we tested the Ti_50−x_V_25_Cr_25_Zr_x_ (x = 0 and 7) alloys. The alloys were thoroughly activated and were in a saturated hydrogen-absorption state. The hydrogen desorption testing was conducted in a closed sample chamber, with a heating-up rate of 10 K/min from the room temperature to 627 K. Figure 6 displays the results. The dehydrogenation equilibrium of the Ti_50_V_25_Cr_25_ alloy was reached in about 1200 s, while the Ti_43_V_25_Cr_25_Zr_7_ alloy was in about 1000 s. For Ti_45_V_25_Cr_25_Zr_5_ alloy and Ti_41_V_25_Cr_25_Zr_9_ alloy, the time to reach dehydrogenation equilibrium was 940 s and 920 s, respectively. The figure shows that the time for the alloy to reach hydrogen release equilibrium decreases with increasing Zr content. This is consistent with hydrogen-absorption kinetics, indicating that the presence of a second phase can increase channels for hydrogen atom diffusion and improve hydrogen-release kinetics. Calculations revealed that the hydrogen-desorption ratio of the Ti_43_V_25_Cr_25_Zr_7_ alloy was 47%, while that of the Ti_50_V_25_Cr_25_ alloy was only 43%. The data indicate that the hydrogen-desorption ratio improved in the alloy with Zr addition. It can be observed from the figure that, as the reaction progresses, the hydrogen pressure in the sample chamber increases, allowing hydrogen atoms to enter the interior of the alloy to generate a solid solution. The de-/hydriding processes in the alloy eventually reach a dynamic equilibrium, resulting in incomplete hydrogen desorption.

### 3.3. PCT Curve Analyze

**Figure 7:**
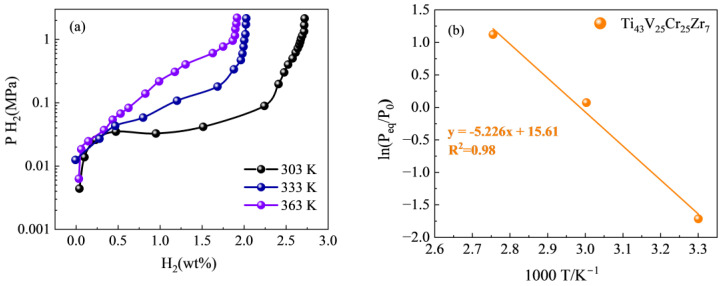
(**a**) PCT curves of as-cast Ti_43_V_25_Cr_25_Zr_7_ alloy at different temperatures; (**b**) Van’t Hoff plots of Ti_43_V_25_Cr_25_Zr_7_ alloy.

In order to investigate the effect of the Zr element addition on the thermodynamic properties of the alloy samples, hydrogen absorption PCT curves of the excellent kinetics Ti_43_V_25_Cr_25_Zr_7_ alloy were conducted at 303 K, 333 K, and 363 K (Figure 7). The resulting curves can be used to plot the Van’t Hoff curve and calculate the enthalpy and entropy of hydrogen absorption, providing a valuable tool for evaluating the thermodynamic characteristics of the alloy. Figure 7a illustrates that both the alloy α-phase (TiV) and β-phase (V_2_H) mutual solubility and the alloy plateau pressure increase with temperature increase. As a result, the slopes of the curves increase while their width and reversible hydrogen storage decrease, which suggests an increase in the critical pressure required for the transition of Zr to the metal hydride phase. The distance between the metal and hydrogen atoms increases, leading to a reduced binding energy between them [38]. Therefore, Zr-added alloys demonstrate greater plateau pressures owing to the existence of more unstable hydrides.

According to the Van’t Hoff equation:

(1) $$\ln\frac{P_{eq}}{P_{\theta }}=\frac{∆H}{RT}-\frac{∆S}{R}$$

In the equation, *P_eq_* represents the equilibrium pressure at various temperatures, while *P_θ_* is the atmospheric pressure, and *R*, *T*, Δ*H*, and Δ*S* are the universal gas constant, thermodynamic temperature, enthalpy change of metal hydrides, and entropy change, respectively. The enthalpy and entropy change of the alloy are calculated by fitting the Van’t Hoff curve with the slope (Δ*H*/*R*) and the intercept (Δ*S*/*R*), respectively. Figure 7b shows the Van’t Hoff curve of the Ti_43_V_25_Cr_25_Zr_7_ alloy, and the fitting effect is good. It can be seen from the figure that the Ti_43_V_25_Cr_25_Zr_7_ alloy has an Δ*H* of −43.45 kJ/mol H_2_ and an Δ*S* of −129.78 J/K/mol H_2_. The absolute value of enthalpy of the Ti_43_V_25_Cr_25_Zr_7_ alloy is lower than that of the Ti_50_V_25_Cr_25_ alloy by −64.4 kJ/mol H_2_ [39]. The Zr substitution alloys reduce the stability of the metal hydride and lower the hydrogen release temperature, and the enthalpy value of these alloys is within the range of the actual application limits of 20~50 kJ/mol H_2_.

### 3.4. DSC

**Figure 8:**
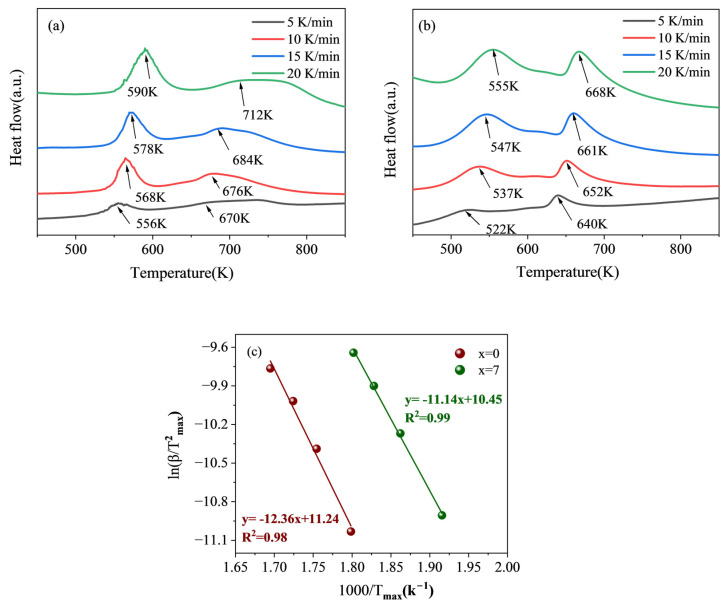
(**a**,**b**) DSC curve of Ti_50−x_V_25_Cr_25_Zr_x_ (x = 0 and 7): (**a**) x = 0, (**b**) x = 7, (**c**) dehydrogenation Kissinger curves for the as-cast Ti_50−x_V_25_Cr_25_Zr_x_ alloys.

As shown in Figure 8a,b, the DSC curves of Ti_50_V_25_Cr_25_ alloy and Ti_43_V_25_Cr_25_Zr_7_ alloy were investigated at heating rates of 5 K/min, 10 K/min, 15 K/min, and 20 K/min. Both alloys exhibited two hydrogen desorption peaks, with the peak temperature and position drifting significantly with increasing heating rates. Taking 10 K/min as an example, the dehydrogenation temperature of the Ti_50_V_25_Cr_25_ alloy was 568 K, while the Ti_43_V_25_Cr_25_Zr_7_ alloy dehydrogenated at 537 K. Thus, it can be inferred that the addition of Zr decreases the onset temperature, which can be attributed to the destabilization of the hydride [40]. The alloy with added Zr showed a lowered starting temperature, indicating that the Ti_43_V_25_Cr_25_Zr_7_ alloy is more conducive to the hydrogen release reaction than the Ti_50_V_25_Cr_25_ alloy. It has been reported that the transformation of the monohydride compound occurs between 500 and 600 K, indicating that the endothermic peak around 550 K corresponds to the phase transition temperature of the monohydride compound. The second endothermic peak occurring at 670 K represents the transition from the hydrogen-containing BCC phase to the hydrogen-free BCC phase [41]. Since the literature indicates that the decomposition temperatures of the ZrV_2_H_x_ and ZrH_1.66_ phases are between 550 and 670 K, it is suggested that the endothermic peak at 650 K corresponds to the ZrVH_1.18_ phase [42].

The dehydrogenation activation energy of an alloy can be determined using the Kissinger equation by analyzing the differential scanning calorimetry curves at various heating rates:

(2) $$\ln(\frac{\beta }{T_{max}^{2}})=-\frac{E}{{RT}_{max}}+\ln\left(k_{0}\right)$$

where *β*, *T_max_*, and *k*_0_ represent the heating rate of the alloy, the peak temperature of the alloy at different temperatures, and a constant, respectively. The fitting is performed with $\ln(\frac{\beta }{T_{max}^{2}})$ as the ordinate, and 1/*T_max_* as the abscissa. The dehydrogenation activation energy of the alloy can be obtained by calculating the slope, as seen in Figure 8c. The value of the dehydrogenation activation energy of the Ti_43_V_25_Cr_25_Zr_7_ alloy is computed to be 92.62 kJ/mol, lower than the 102.67 kJ/mol activation energy of the Ti_50_V_25_Cr_25_ alloy, which can contribute to the increased presence of hydrogen atom diffusion pathways in the presence of the second phase [35].

### 3.5. Cycling and Properties

**Figure 9:**
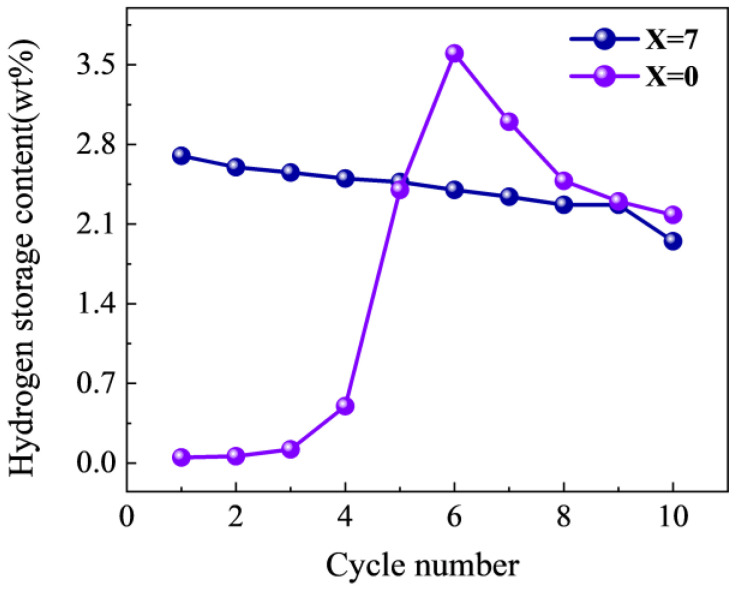
Hydrogen capacities of Ti_50−x_V_25_Cr_25_Zr_x_ (x = 0 and 7) alloys over 10 cycles at 303 K.

The cyclic performance of alloys is one of the criteria for judging whether an alloy is suitable for application. Therefore, the cyclic performance of the alloy was evaluated by hydrogenation at 303 K and 3 MPa and dehydrogenation for 2 h at 673 K. The results are shown in Figure 9. It can be seen from Figure 9 that the Ti_50_V_25_Cr_25_ alloy reached a maximum hydrogen storage capacity of 3.6 wt% after six cycles. This is due to the fact that a nanoscale oxide coating on the surface of the Ti-V-Cr-based alloy is frequently developed which hinders hydrogen atoms from entering the interior of the alloy, thus requiring activation during cycling. The Ti_50_V_25_Cr_25_ alloy reaches its maximum hydrogen uptake after six activations. This is likely due to the alloy producing a clean surface at this point, which facilitates the dissociation of hydrogen molecules into hydrogen atoms on the alloy surface and increases the diffusion rate into the alloy, as suggested by Buzidi et al. [43]. After cycling, the hydrogen-absorption capacity decreased to 2.18 wt%, with a cycle retention rate of only 60% (four cycles). The Ti_43_V_25_Cr_25_Zr_7_ alloy demonstrated excellent cyclic stability, with a maximum hydrogen-absorption capacity of 2.7 wt% in the first cycle and a decrease to 1.95 wt% after ten cycles, with a cycle retention rate of ~72%. Priyanka Ruz [39] pointed out that the cyclic performance of Ti-V-Cr alloys is poor under oxygen, even at low oxygen conditions, as these alloys are susceptible to oxide formation. On the other hand, zirconium demonstrates a greater affinity for oxygen. In the Ti_43_V_25_Cr_25_Zr_7_ alloy, Zr reacts preferentially with oxygen, decreasing the impact of oxygen poisoning on the BCC phase. Hence, the cyclic performance of the Ti_43_V_25_Cr_25_Zr_7_ alloy is superior to that of the Ti_50_V_25_Cr_25_ alloy.

**Figure 10:**
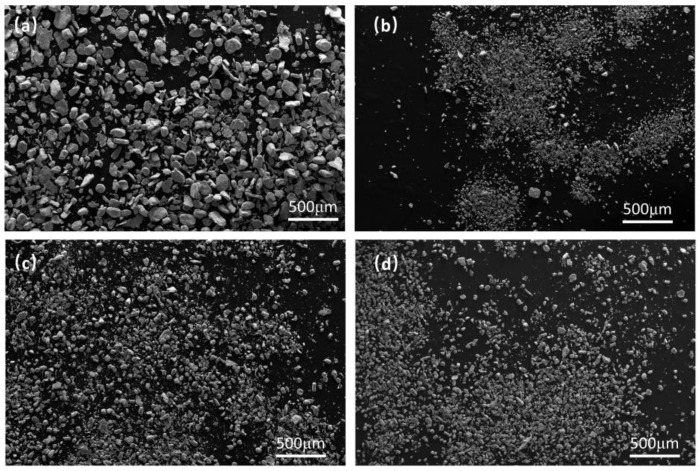
Particle distribution before and after cycling of Ti_50−x_V_25_Cr_25_Zr_x_ alloys: (**a**) as-cast (x = 0); (**b**) after 10 de-/hydrogenation cycles (x = 0). (**c**) as-cast (x = 7); (**d**) after 10 de-/hydrogenation cycles (x = 7).

In addition to the presence of impurities, the cyclic performance of the alloy is also affected by the particle size of the powder. Figure 10 shows a comparison of the size before and after alloy cycling. It is evident that the powder of the Ti_50_V_25_Cr_25_ alloy exhibits distinct pulverization after cycling, whereas the Ti_43_V_25_Cr_25_Zr_7_ alloy is almost constant for successive cycles. This result indicates that the addition of Zr can stabilize the crystal structure after hydrogen absorption and desorption contraction and implies there is a certain relationship between cyclic performance and pulverization degree.

## 4. Conclusions

In this work, four alloys with different Zr contents were prepared by arc melting. The hydrogen storage capacity, kinetics, and activation performance of the alloys were studied. The results indicate that the Ti_50−x_V_25_Cr_25_Zr_x_ (x = 0, 5, 7, and 9) alloys exhibit the C15 Laves phase and the abundance gradually increases with the increase in Zr content, while the abundance of the BCC phase decreases. The addition of the Zr element enhances the activation and kinetic performance of the alloy, and the hydrogen absorption initially increases and then decreases. The hydriding enthalpy with −43.45 kJ/mol H_2_, and the activation energy with 92.62 kJ/mol, of the Ti_43_V_25_Cr_25_Zr_7_ alloy are lower than those of the Ti_50_Cr_25_V_25_ alloy. The Ti_43_V_25_Cr_25_Zr_7_ alloy has a better cycle stability than that of the Ti_50_Cr_25_V_25_ alloy. After ten cycles, the hydrogen absorption decreases from the initial 2.7 wt% to 1.95 wt%, with a retention rate of ~72%. This study provides information on the influence of different Zr contents on the microstructure and hydrogen storage properties of Ti_50_Cr_25_V_25_. In the future, we can further investigate the influencing factors of Zr doping, as well as the influencing mechanism of the BCC phase and Laves phase on the hydrogen storage performance, which can provide better guidance for the design of hydrogen storage compositions.

## Figures and Tables

**Table 1:** Rietveld refinement results of XRD of as-cast Ti_50−x_V_25_Cr_25_Zr_x_ (x = 0 and 7) alloys.

X	R_w_	Phase	Lattice Parameter (Å)	Phase Abundance (wt%)
X = 0	8.2	BCC	3.128 (0)	100
X = 7	6.3	BCC	3.133 (8)	85.9
		C15 Laves	7.288 (8)	14.1

**Table 2:** Summary of EDS as-cast Ti_50−x_V_25_Cr_25_Zr_x_ alloys (at.%).

Zr Content	Phase	Compositions (at%)			
		Ti	V	Cr	Zr
X = 0	BCC	50.93	24.7	24.36	-
X = 5	BCC	46.17	26.69	24.43	2.69
	C15 Laves	38.67	17.5	25.68	18.13
X = 7	BCC	43.82	27.28	25.75	3.15
	C15 Laves	39.17	17.08	24.72	19.42
x = 9	BCC	41.46	30.14	25.40	3.3
	C15 Laves	34.75	17.96	26.69	20.61

## Data Availability

Data are contained within the article and Supplementary Materials.

## Supplementary Materials

The following supporting information can be downloaded at: https://www.mdpi.com/article/10.3390/ma17061366/s1. Figure S1. Element mapping of as-cast Ti_45_V_25_Cr_25_Zr_5_ alloy.
