# Supplementary material for: Influence of Zr Addition on the Microstructure and Hydrogenation Kinetics of Ti50−xV25Cr25Zrx (x = 0, 5, 7, and 9) Alloys
Source: Materials (Basel). 2024 Mar 16;17(6):1366. doi: 10.3390/ma17061366 (PMC10972261; doi:10.3390/ma17061366)
Supplement: Supplementary file 1 [file materials-17-01366-s001.zip › materials-2894980-supplementary.pdf]

materials

Type of the Paper (Article, Review, Communication, etc.)

Influence of Zr addition on the microstructure and hydrogenation kinetics of $Ti_{50-x}V_{25}Cr_{25}Zr_x$ (x=0, 5, 7, and 9) alloys

Qianying Zeng [1], Feng Wang [1,*], Zhengxi Li[1], Maohua Rong[1,*] Jiang Wang[1], and Zhongmin Wang[2]

1 School of Materials Science and Engineering, Guilin University of Electronic Technology, Guilin 541000, China;
2 Guangxi Academy of Sciences, Nanning 530007, China;
* Correspondence: wf@guet.edu.cn or rongmh124@guet.edu.cn

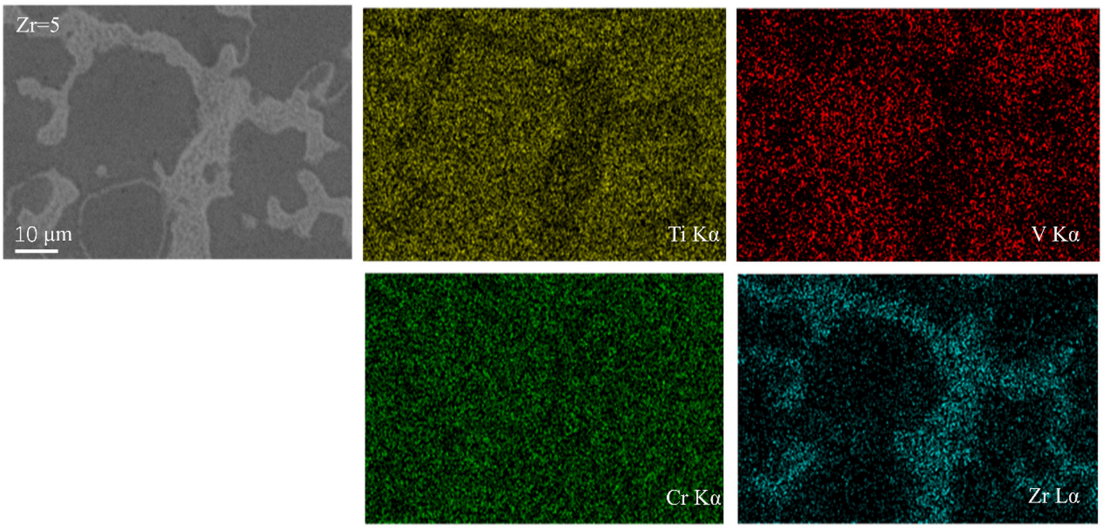

Figure S1. Element mapping of as-cast $Ti_{45}V_{25}Cr_{25}Zr_5$ alloy.
